# Supplementary material for: A conserved RNA structural motif for organizing topology within picornaviral internal ribosome entry sites
Source: Nat Commun. 2019 Aug 9;10:3629. doi: 10.1038/s41467-019-11585-z (PMC6689051; doi:10.1038/s41467-019-11585-z)
Supplement: Supplementary file 1 — Supplementary Information [file 41467_2019_11585_MOESM1_ESM.pdf]

Supplementary Information

A conserved RNA structural motif for organizing topology within picornaviral internal ribosome entry sites

Koirala et al.

Supplementary Note 1

Figure 3c provides an overall schematic of the interactions in the Fab-RNA binding interface. Beginning with the P1a stem region at the base of the bulge L1, the side chain functional groups of the CDR L1 residues, S29 and Y31, and the scaffold residue, R67, interact with the phosphodiester backbone on the 5'-side of the P1.1 stem (Figure 3c, Supplementary Figure 11). The amino and hydroxyl groups at S29 interact with the phosphates at C602 and A603, respectively; the R67 guanidinium group interacts with the phosphates at A603 and U604; and the Y31 hydroxyl group interacts with the phosphate at U605. In addition, S29 contributes to a water-mediated network of hydrogen bonds involving the A603 and U604 nucleobases (Figure 3c and Supplementary Figure 11). Continuing upwards along the P1 helical axis, three consecutive residues R93, R94 and R95 from CDR L3 interact mainly with the flipped-out nucleotide U674 at the 3'-side of the L12 (Figure 3c, d). Consistent with the U674 interactions observed in the crystal structure, the deletion construct, HAV-ΔU674, binds to HAVx with lower affinity (K_d = 176 ± 18 nM) compared to the parent dV construct (Supplementary Figure 10). R93 and R95 form direct hydrogen bonds to the nucleobase; R94 forms a direct hydrogen bond to the 2'-OH and together with R95 forms a water-mediated hydrogen bond to the nucleobase; R93 also forms hydrogen bonds to wobble paired U604 and G675. In the upper region of the asymmetric bulge, heavy chain CDRs H2 and H3 make extensive interactions with the RNA. Three residues from CDR H2, two serines and a tyrosine, interact with the RNA via stacking and hydrogen bonding interactions (Figure 3c, e). Y57 stacks on U672 and forms a hydrogen bond with the U671 phosphodiester. Both S55 and S58 interact with the phosphodiesters of A673, and S58 forms two additional hydrogen bonds with U672. CDR H3 utilizes the highest number of residues (six) among all CDRs to interact with the RNA via direct or water-mediated hydrogen bonds and stacking interactions. To insert into a pocket formed within CDR-H3, A673 projects towards the interior of the helix, accommodated by a water-mediated hydrogen bonding network involving U606 and U671 (Figure 3a, c, f). Consistent with the extensive interactions of U672 with the Fab, the deletion mutation, HAV-ΔU672, severely attenuates Fab affinity (K_d > 1000 nM, Supplementary Figure 10). Y104 stacks with U672, Y109 with U605 and H105 with G631. Hydrogen bonding interactions occur between Y104 and U671, H105 and A673, Y109 and A673, Y110 and U605, and between W111 and the phosphodiester of A673.

Supplementary Note 2

Known RNA binding proteins that recognize bulges include zinc-finger domains,[1,2] L7ae kink turn-binding proteins,[3] and HIV-1 Tat.[4] The zinc-finger domain structure consists of a β-hairpin and an α-helix stabilized by Zn^{2+} and primarily uses α-helix residues to recognize the bulged nucleotides. The mode of recognition includes stacking of aromatic protein side chains with bulged RNA bases, electrostatic interactions of positively charged protein side chains with the ribose-phosphate backbone of the RNA and direct and water-mediated hydrogen bonding between the protein side-chain and main chain atoms with either the RNA backbone or the bases.

The L7ae class of proteins binds to the kink-turn – a bulged RNA motif.[3] The L7ae RNA-binding motif consists of a highly basic β-strand, an α-helix and a short loop of hydrophobic residues. The binding interface involves hydrogen bonding, stacking, electrostatic and hydrophobic interactions.

The recently reported structures of 7SK snRNA and HIV-1 TAR RNA in complex with Tat protein's RNA binding domain have revealed that the domain mainly interacts with an RNA bulge[4] via a so-called arginine sandwich motif (ASM). The ASM intercalates into the RNA bulge, and the bulge nucleotides stack in a sandwich-like manner above and below the guanidinium moiety of an arginine residue. In general, RNA recognition involves stacking, electrostatic and hydrogen bonding interactions.

In contrast to these natural RNA binding proteins, the immunoglobulin fold of Fab HAVx contains a beta barrel with projecting CDR loops that create a relatively smooth surface for recognizing different structural features of the L1 bulge in HAV dV. The mode of RNA recognition by Fab HAVx includes stacking of aromatic protein side chains with bulged RNA bases, electrostatic interactions of positively charged protein side chains with the ribose-phosphate backbone of the RNA and direct and water-mediated hydrogen bonding between the protein side-chain and main chain atoms with either the RNA backbone or the bases.

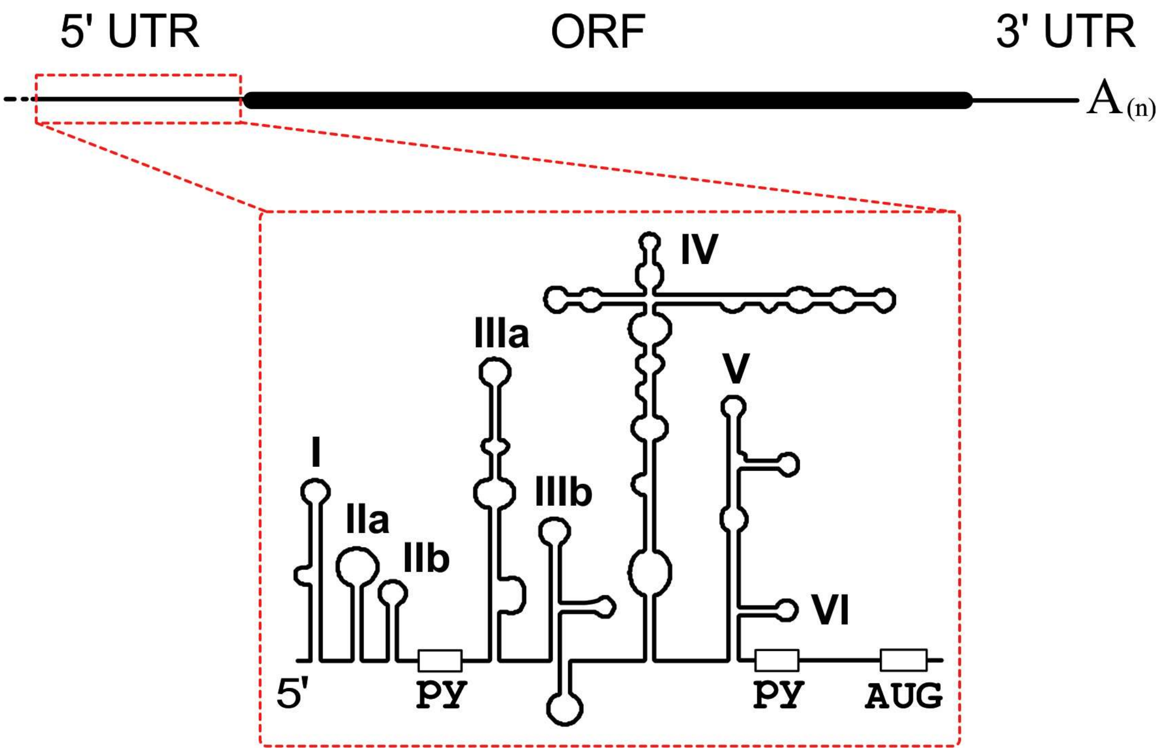

Supplementary Figure 1: Overall schematic of the HAV genome. The ~7.5 kb positive-sense ssRNA genome consists of a single open reading frame (ORF) flanked by 5' and 3' UTRs. The 5'-UTR is highly conserved among HAV strains and clinical isolates.[5] It contains six modular domains (labelled I through VI) comprising highly organized secondary structures that include the IRES elements. AUG and py indicate the initiation codon and the polypyrimidine tract, respectively.

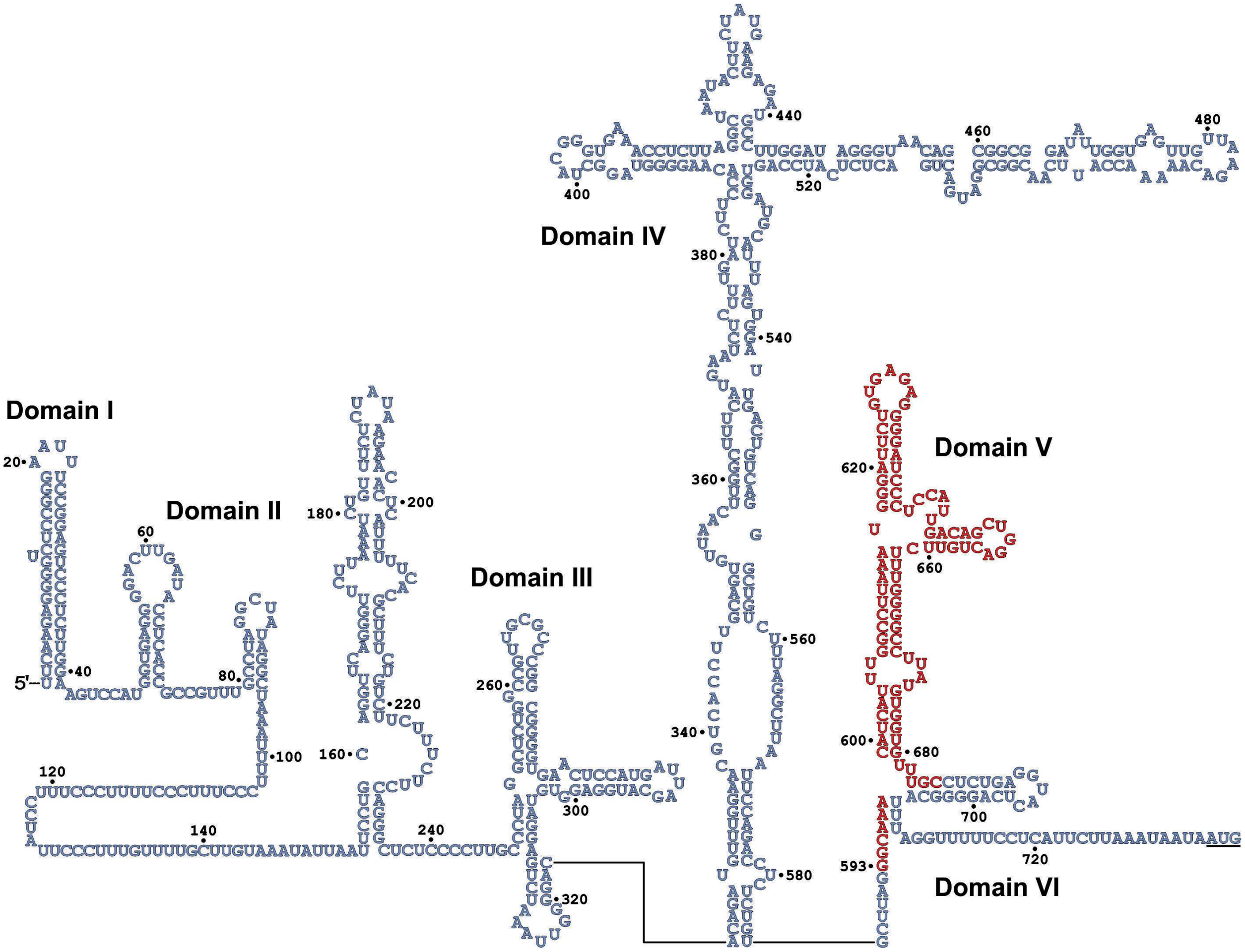

Supplementary Figure 2: Sequence of the 5'-UTR of the wild-type HAV strain, HM175.[6] The secondary structure of the domains and their nomenclature are shown according to the biochemical probing and co-variation analysis of Brown et. al.[6] Our phage display selection and crystallization construct (highlighted red) includes 92 nucleotides from 593 to 684. In this construct, nucleotides C595-A598 are expected to form base pairs with U681-G684 to continue the 5'-3' helical stem.

a.

CDR-L1 (29-33)	CDR-L2 (51-54)	CDR-L3 (92-97)
SVYYS	SASY	YRRRPI

CDR-H1 (31-37)	CDR-H2 (53-62)	CDR-H3 (102-114)
SLSSSSM	SISPYSGSTS	TKYHYKNYYWWAL

b.

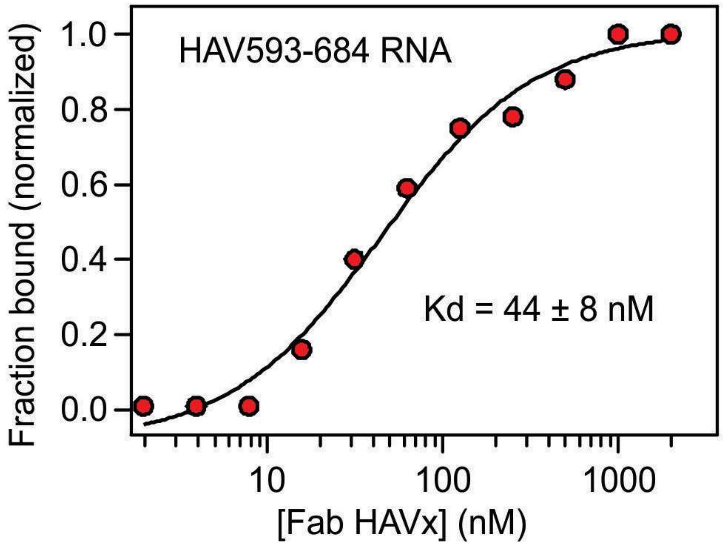

c.

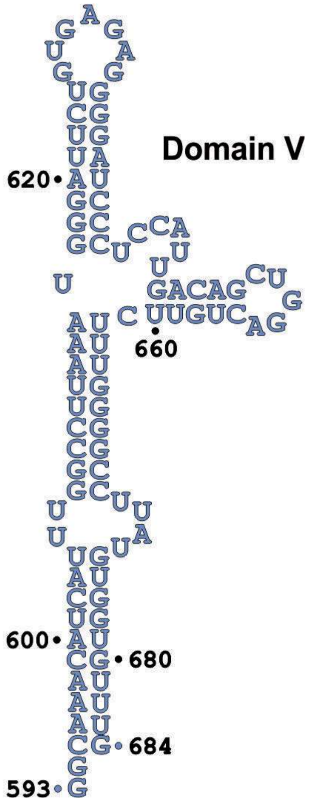

Supplementary Figure 3: Fab HAVx binding affinity against the dV crystallization construct. (a) Residues from CDRs of the Fab HAVx. (b) A representative plot of fraction bound as a function of varying concentration of Fab HAVx for the dV RNA construct as accessed from filter binding assay in 10 mM tris, pH 7.5, 50 mM KCl, 10 mM $MgCl_2$ buffer at 23°C. The reported K_d (44 ± 8 nM) is the value (average ± standard deviation) obtained from ≥ 3 independent measurements. (c) HAV593-684 (or dV) RNA construct.

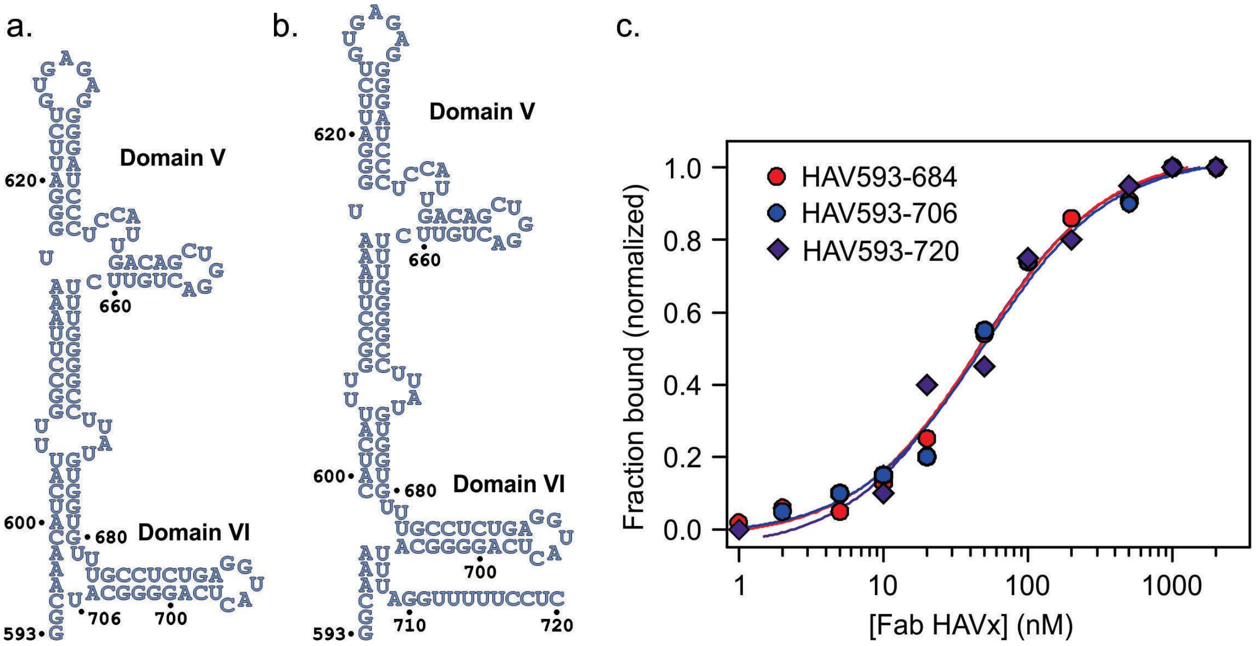

Supplementary Figure 4: Fab HAVx binding affinity for the RNA constructs HAV593-706 (a) and 593-720 (b). (c) Plots of fraction bound as a function of varying concentration of Fab HAVx for HAV593-684 (red), HAV593-706 (blue) and HAV593-720 (magenta) constructs as accessed from filter binding assays in 10 mM tris, pH 7.5, 50 mM KCl, 10 mM $MgCl_2$ buffer at 23°C. K_ds obtained for HAV593-706 and HAV593-720 against the Fab HAVx are 49 ± 8 nM, 47 ± 6 nM, respectively. K_ds are the values (average ± standard deviation) obtained from ≥ 3 independent measurements.

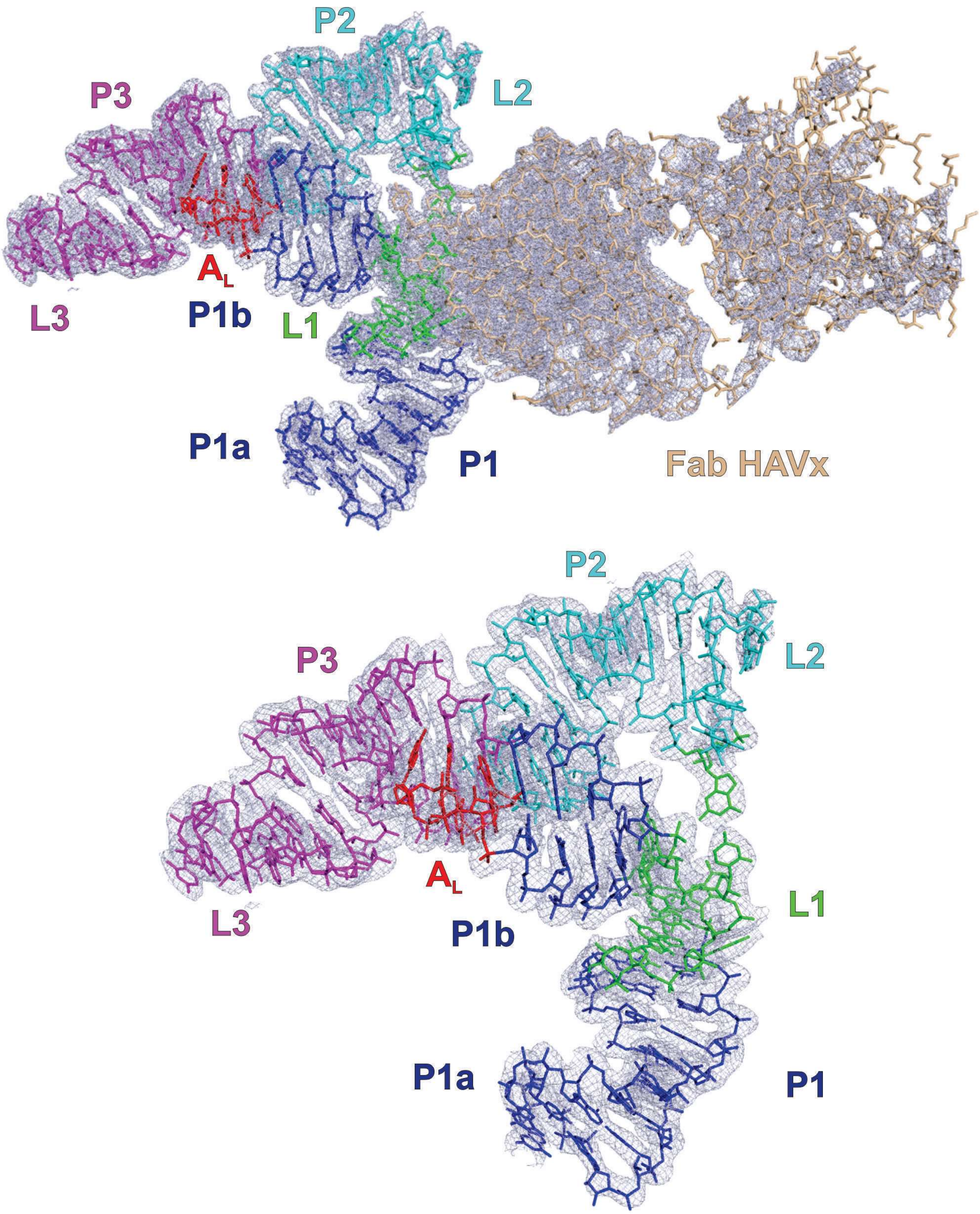

Supplementary Figure 5: Crystal structure of HAV IRES dV RNA in complex with Fab HAVx solved at 2.84-Å resolution. Blue mesh represents the $2|F_o| - |F_c|$ electron density map at 1σ contour level and carve radius 1.8 Å.

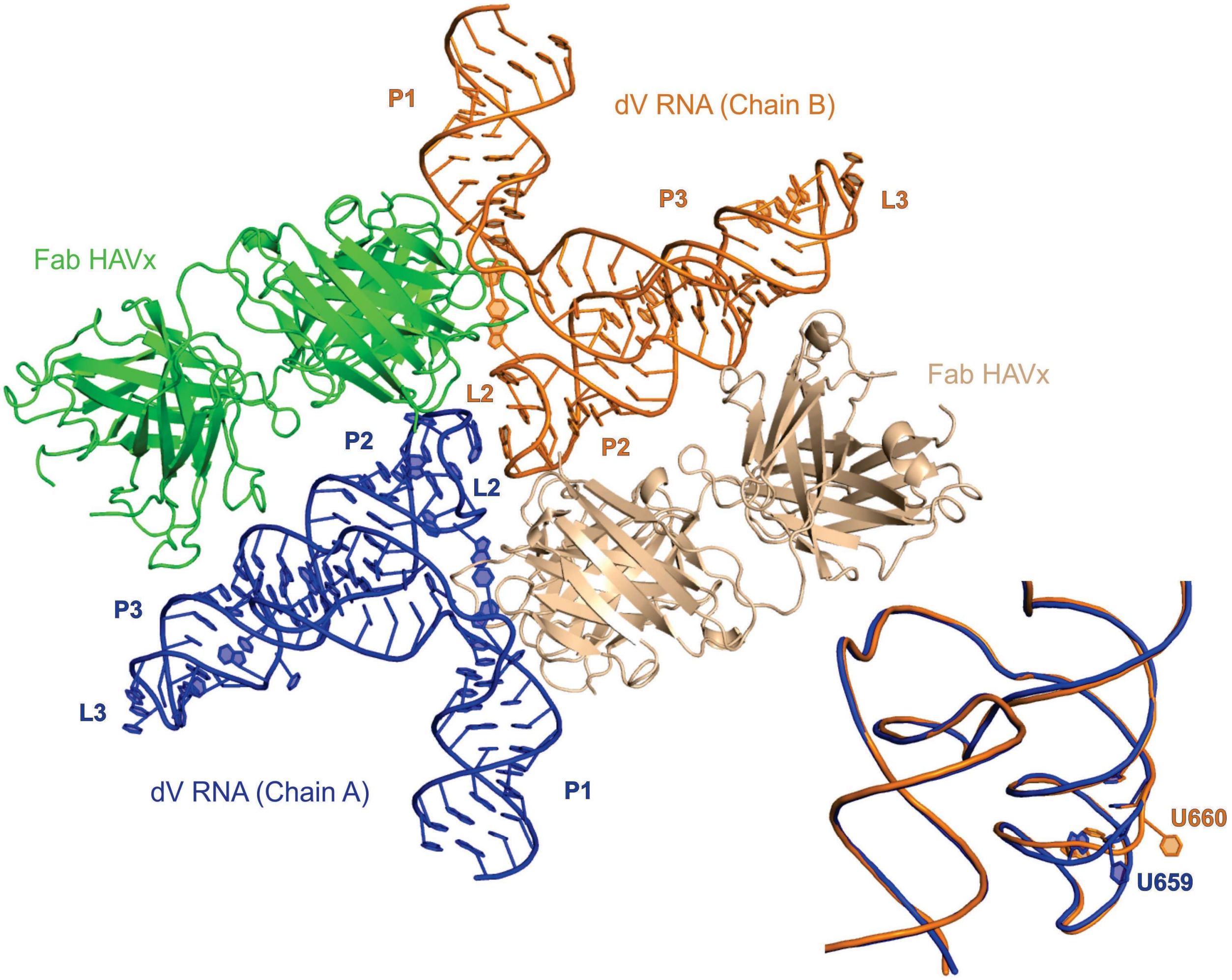

Supplementary Figure 6: Crystals of the Fab-RNA complex contain two molecules of the complex per asymmetric unit. For convenience, two RNA molecules are designated as chain A (blue) and chain B (orange). Fabs in complex with chain A and chain B are colored wheat and green, respectively. The two RNA chains are identical except that U659 in chain A and U660 in chain B are unpaired and flipped out of the helix. The nucleotides U660 and U659 remain base-paired in A and B chains, respectively.

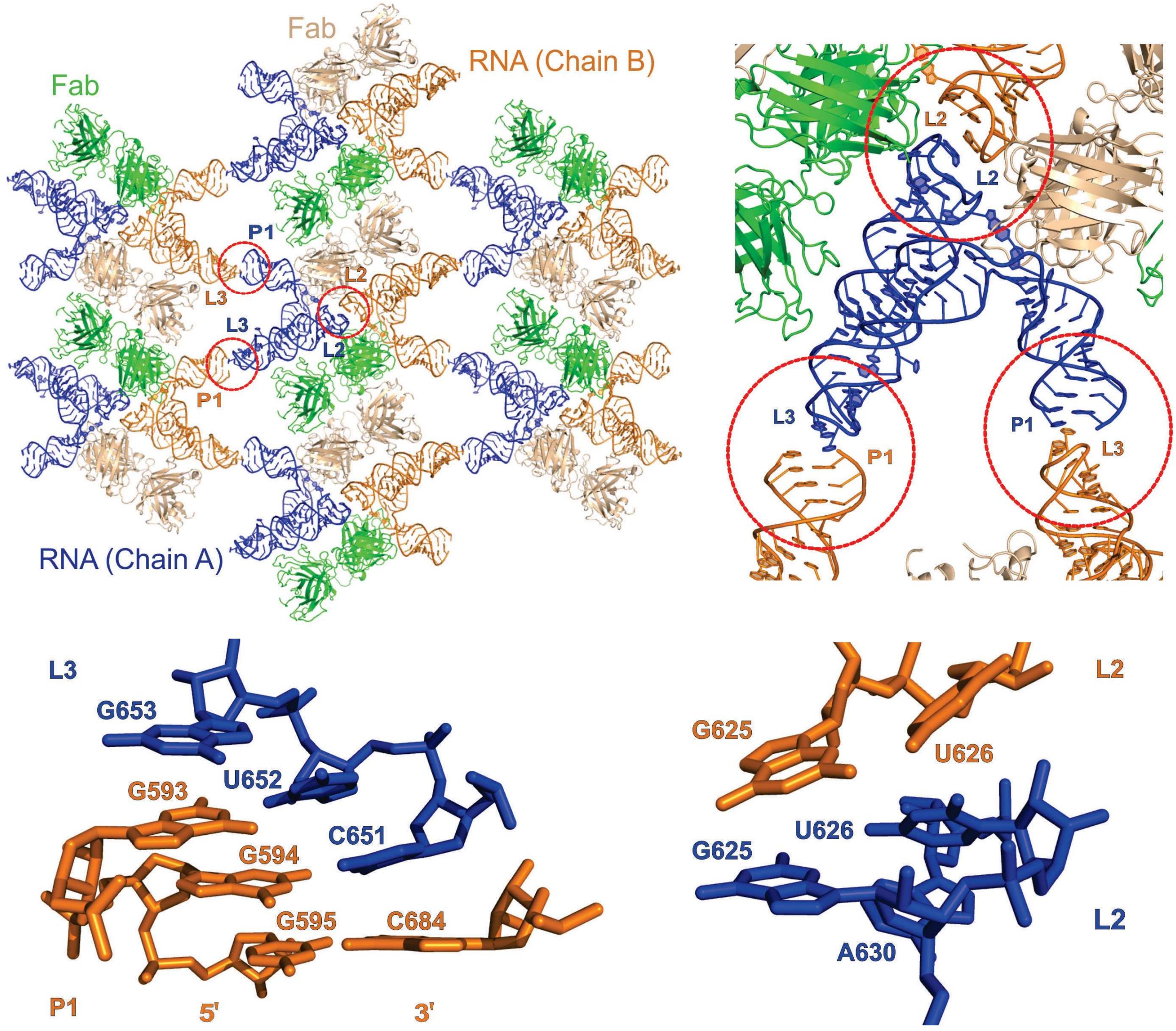

Supplementary Figure 7: Crystal packing of the Fab-RNA complex and corresponding Fab-Fab, Fab-RNA and RNA-RNA interfaces. Fab-Fab and Fab-RNA contacts including the binding interface account for ~32% and ~50%, respectively of the total crystal contacts. The Fab-Fab interactions within the lattice take place between the outer surfaces of the both heavy and light chain constant domains. RNA-RNA interactions account for ~18% of the crystal contacts. Each RNA molecule makes crystal contacts with its three neighboring RNA molecules. Two sets of these crystal contacts involve base pairing interactions between loop L3 and the helix P1. The third RNA – RNA crystal contact occurs between L2 loops from neighboring molecules, involving stacking interactions of nucleotides G625 and U626. Other interactions between symmetry related L2 loops were ambiguous due to poor electron density for the reliable modeling of the nucleotides G627-G629.

Supplementary Table 1: SAXS data collection and analysis statistics.

**Data collection**			**Software**	
Instrument	SIBYLS beamline Advanced Light Source (ALS) Berkeley, CA		Data merging	ALMERGE[7]
Beam geometry	Superbend Synchrotron Light 100 x 100 micron		Model intensity curve fitting	FOXS[8]
Wavelength (Å)	1.27		Guinier analysis	AUTORG[9]
Q range ($Å^{-1}$)	0.012 – 0.54		P(r) distribution calculation	DATGNOM[10]
Exposure time / slicing time (s)	10 / 0.3		*Ab initio* molecular envelopes	DAMMIF[11]
Temperature (K)	283		Structure superposition	SUPCOMB[12]
**Data analysis**				
**Fab HAVx – dV complex**			**Standalone dV**	
	SAXS	Crystal structure	SAXS	Crystal structure
Rg Guinier fit (Å)	35.6 ± 0.4	34.8	27.8 ± 0.1	23.9
Rg P(r) distribution (real space) (Å)	37.0		28.2	
Rg P(r) distribution (reciprocal space) (Å)	36.9		28.1	
D_{max} P(r) distribution (Å)	125.0	130.7	97.2	70.0
I_0 Guinier fit (AU)	70.5 ± 0.3		56.5 ± 0.1	
I_0 P(r) distribution fit (AU)	71.2 ± 0.2		56.3 ± 0.1	

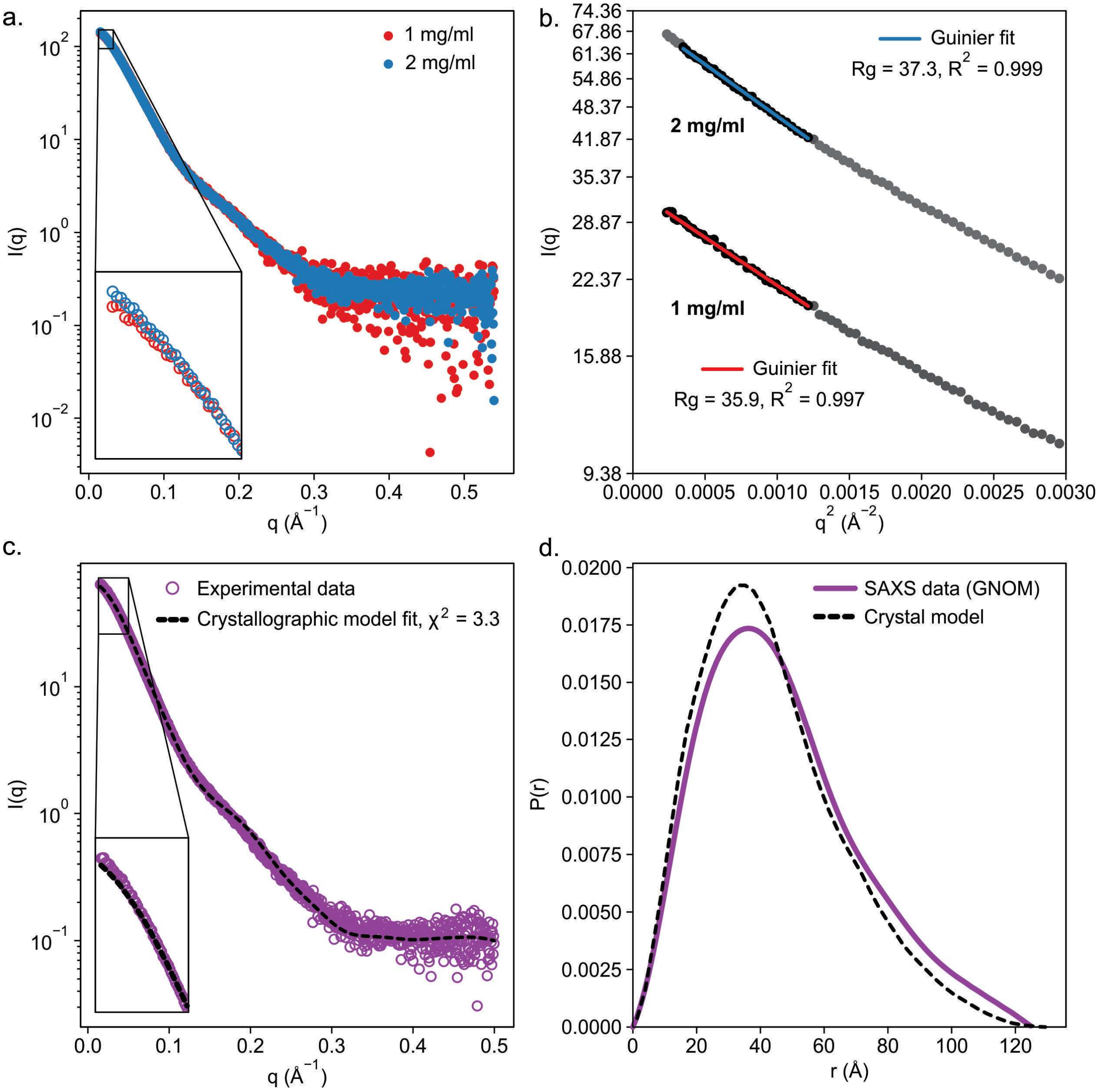

Supplementary Figure 8: Solution phase SAXS analysis of the dV RNA in complex with HAVx Fab. (a) SAXS datasets from two different concentrations of the Fab-RNA complex, consisting of 1.0 mg/ml (red) and 2.0 mg/ml (blue) RNA with 1:1 molar equivalent of the Fab. As the two show good agreement except at very low q, the 1.0 mg/mL data was scaled to the 2.0 mg/ml intensities and a merged curve generated using ALMERGE.[7] The merged dataset was used for subsequent analysis and is shown in (c). (b) Guinier fitting for R_g determination in the two experimental datasets using AUTORG.[9] The Guinier region is linear (and thus well-behaved) at both concentrations, yielding Rg estimates of 35.9 ± 0.3 and 37.3 ± 0.2 Å for the 1.0 and 2.0 mg/mL samples, respectively. (c) The fit of the merged experimental profile (magenta) to a theoretical

scattering profile calculated using FOXS[8] from the crystal structure atomic coordinates (black). The fit was performed over the range $0.01533 < q < 0.5$ Å^{-1}. The reported χ^2 for the fit is 3.30 (d) Comparison of pairwise-distance distribution [P(r)] curves calculated from the merged experimental (magenta) data using DATGNOM[10] compared to the crystal structure atomic coordinates (black). A summary of the SAXS data collection, analysis software and statistics are provided in Supplementary Table 1.

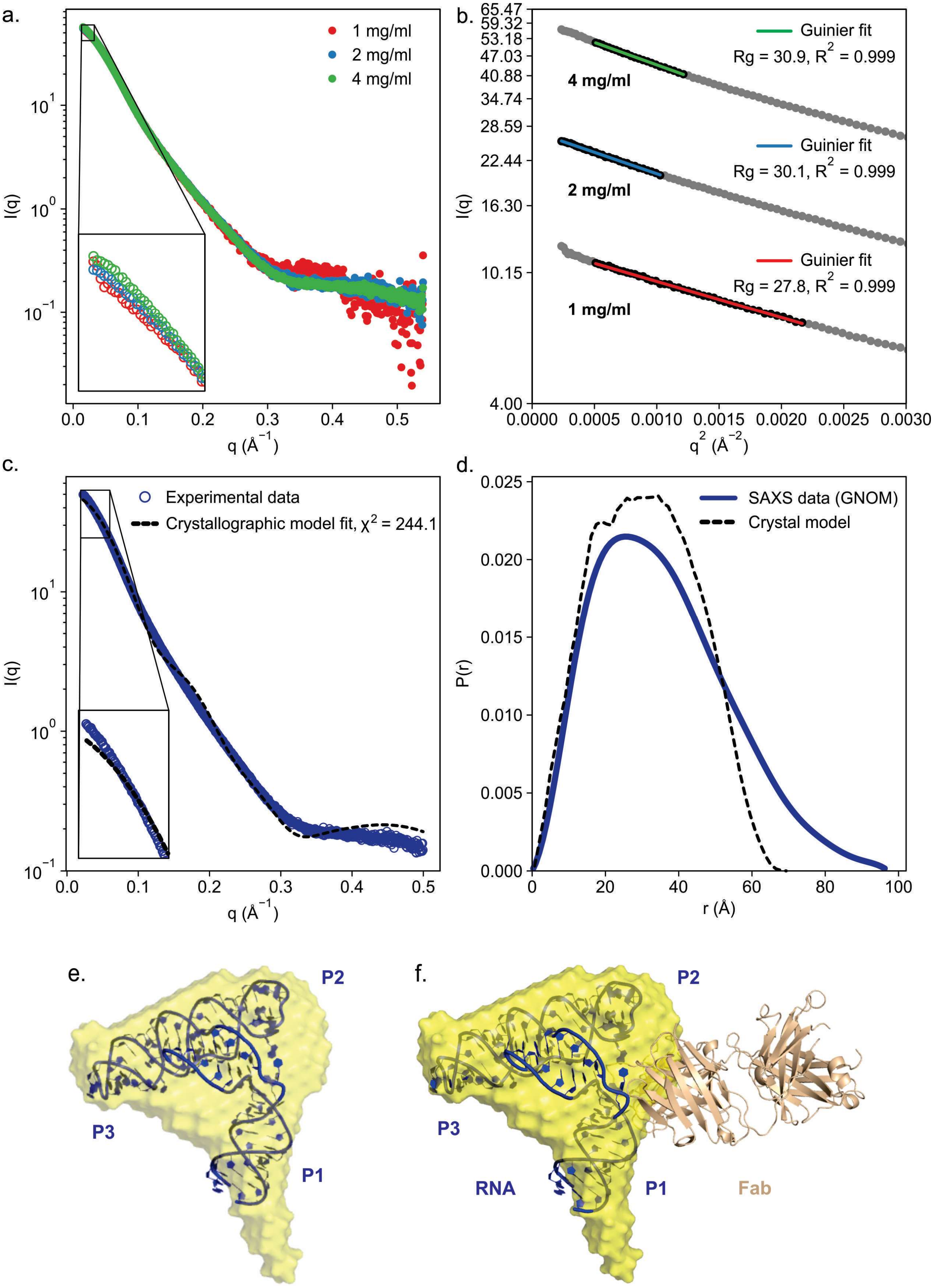
a.
1 mg/ml
2 mg/ml
4 mg/ml
I(q)
q (Å^{-1})
b.
Guinier fit
Rg = 30.9, R^2 = 0.999
4 mg/ml
Guinier fit
Rg = 30.1, R^2 = 0.999
2 mg/ml
Guinier fit
Rg = 27.8, R^2 = 0.999
1 mg/ml
I(q)
q^2 (Å^{-2})
c.
Experimental data
Crystallographic model fit, χ^2 = 244.1
I(q)
q (Å^{-1})
d.
SAXS data (GNOM)
Crystal model
P(r)
r (Å)
e.
P2
P3
P1
f.
P2
P3
RNA
P1
Fab

Supplementary Figure 9: Solution phase SAXS analysis of standalone dV RNA. (a) SAXS datasets from three different concentrations of RNA, 1.0 mg/ml (red), 2 mg/ml (blue) and 4.0 mg/ml (green) were collected. As with the data for the Fab-RNA complex, there is a good agreement between the scattering profiles across concentrations except at very low q, so ALMERGE[7] was used to combine the low-q data from the 1.0 mg/mL dataset with the high-q data from the 4.0 mg/mL dataset. The merged dataset was used for subsequent analysis and is shown in (c). (b) Guinier fitting (black) for R_g determination from the SAXS datasets using AUTORG.[9] Once again, the Guinier region is linear and well-behaved across concentrations. Fitted R_g values are 27.8 ± 0.1, 30.1 ± 1.1, and 30.9 ± 0.8 for the 1.0, 2.0 and 4.0 mg/mL samples, respectively. (c) The fit of the merged experimental profile (blue) to a theoretical scattering profile calculated using FOXS[8] from the RNA portion of the crystal structure (black). The fit was performed over the range $0.0225 < q < 0.5$ Å^{-1}. The reported χ^2 for the fit is 244.1. (d) Comparison of pairwise-distance distribution [P(r)] curves calculated from the merged experimental (blue) data using DATGNOM[10] compared to the crystal structure atomic coordinates (black). (e) Superposition of the SAXS-derived molecular envelope (yellow) for the dV construct with the corresponding crystal structure (blue) by SUPCOMB.[12] (f) Guided by this superposition, alignment of this dV envelope with the RNA portion of the Fab-RNA crystal structure was performed in PyMol. Summary of the SAXS data collection, analysis software and statistics are provided in Supplementary Table 1.

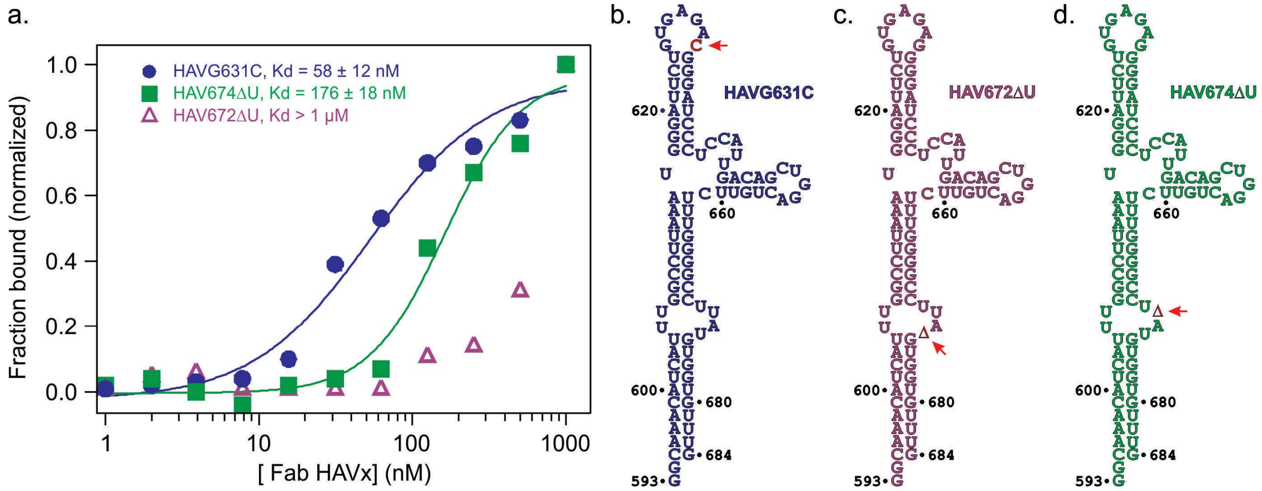

Supplementary Figure 10: Fab HAVx binding affinity to dV mutation and deletion constructs. (a) Plots of fraction bound as a function of varying concentration of Fab HAVx for HAVG631C (blue) (b), HAV672ΔU (green), (c) and HAV674ΔU (purple) (b-d) Secondary structures of these RNA constructs. Data were obtained from filter binding assays in 10 mM tris, pH 7.5, 50 mM KCl, 10 mM $MgCl_2$ buffer at 23°C. The site of mutation or deletion in the RNA constructs in b, c and d are shown in red and also indicated by a red arrow. Note that the G631C mutation in b has the potential to form a C631-G625 base pair, which would extend the P2 helix, and consequently disrupt the interactions of nucleotide 631 with the Fab. Reported K_ds are the values (average ± standard deviation) obtained from ≥ 3 independent measurements.

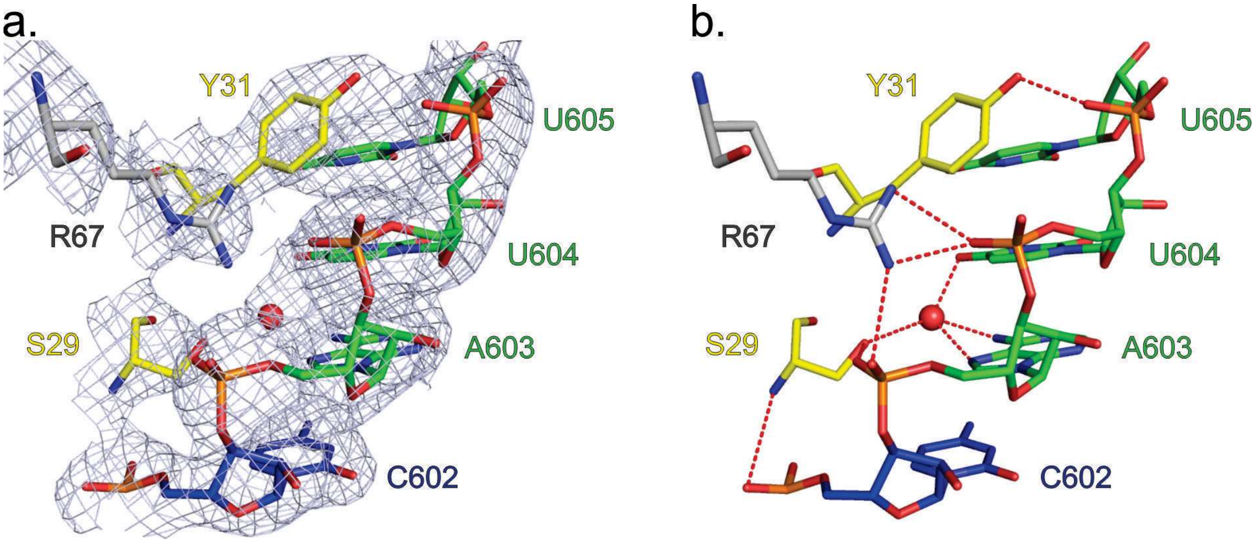

Supplementary Figure 11: Interactions of Fab HAVx CDR-L1 with HAV dV. (a) Two residues, S29 and Y31 from CDR-L1 interacts with C602-U605. Blue mesh represents the $2|F_o|$ - $|F_c|$ electron density map at 1σ contour level and carve radius 1.8 Å. (b) Direct and water (represented by a red sphere) mediated hydrogen bonding network (indicated by red dashed lines reflect heteroatoms within hydrogen bonding distance (2.5 – 3.5 Å) between the residues from CDR L1 (yellow) of the Fab HAVx and the nucleotides (green) within the L1 bulge of the dV RNA. An arginine residue, R67 (gray) is from the light chain's constant domain and C602 nucleotide in blue is from the P1a helix of the dV.

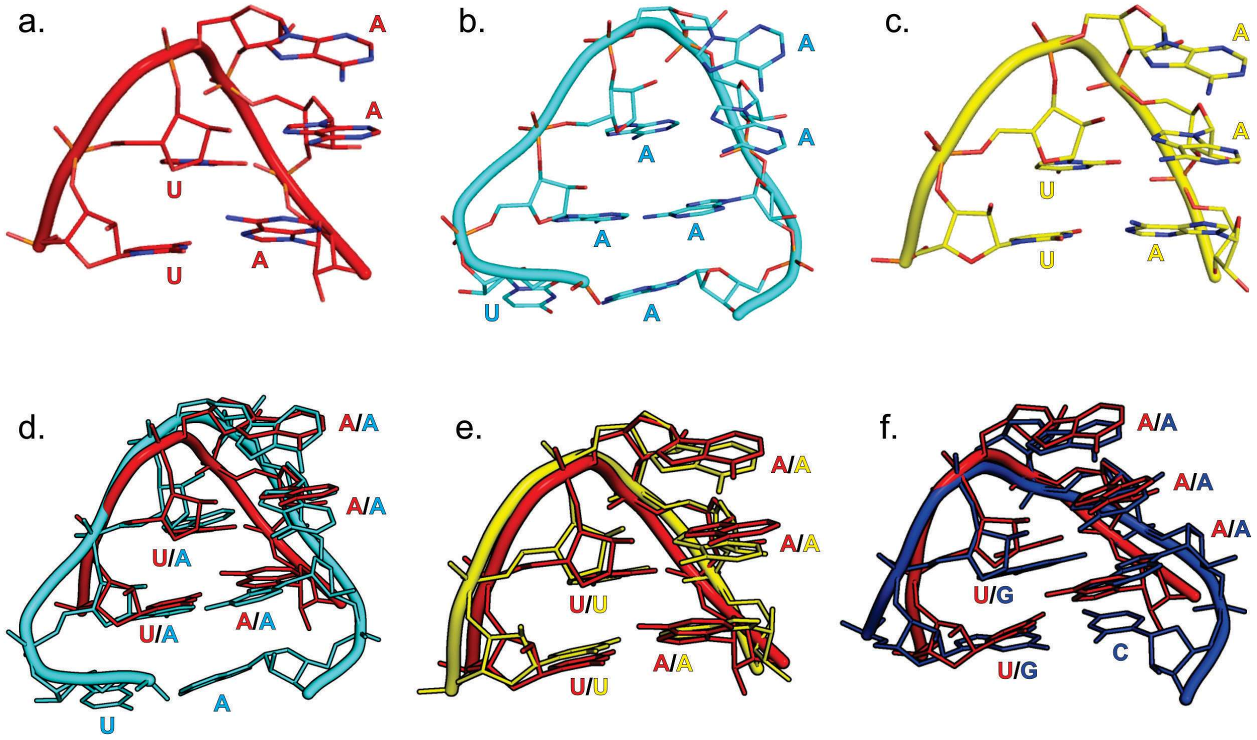

Supplementary Figure 12: Comparison of HAV dV A_L motif with other similar motifs and GNRA tetra-loops. Structures of A_L motif in (a) HAV dV (PDB code: 6MWN), (b) EMCV J-K domain (PDB code: 2NBX)[13] and (c) GTPase associated RNA domain of E. coli 23S rRNA (U1082-A1086, PDB code: 1QA6).[14] (d-f) Superposition of A_L motifs from HAV dV and EMCV J-K domain (d), 23S rRNA U1082-A1086 (e), and a GAAA type GNRA tetraloop observed in the crystal structure of P4-P6 domain of *Tetrahymena* group I intron (f, PDB code: 2R8S).[15]

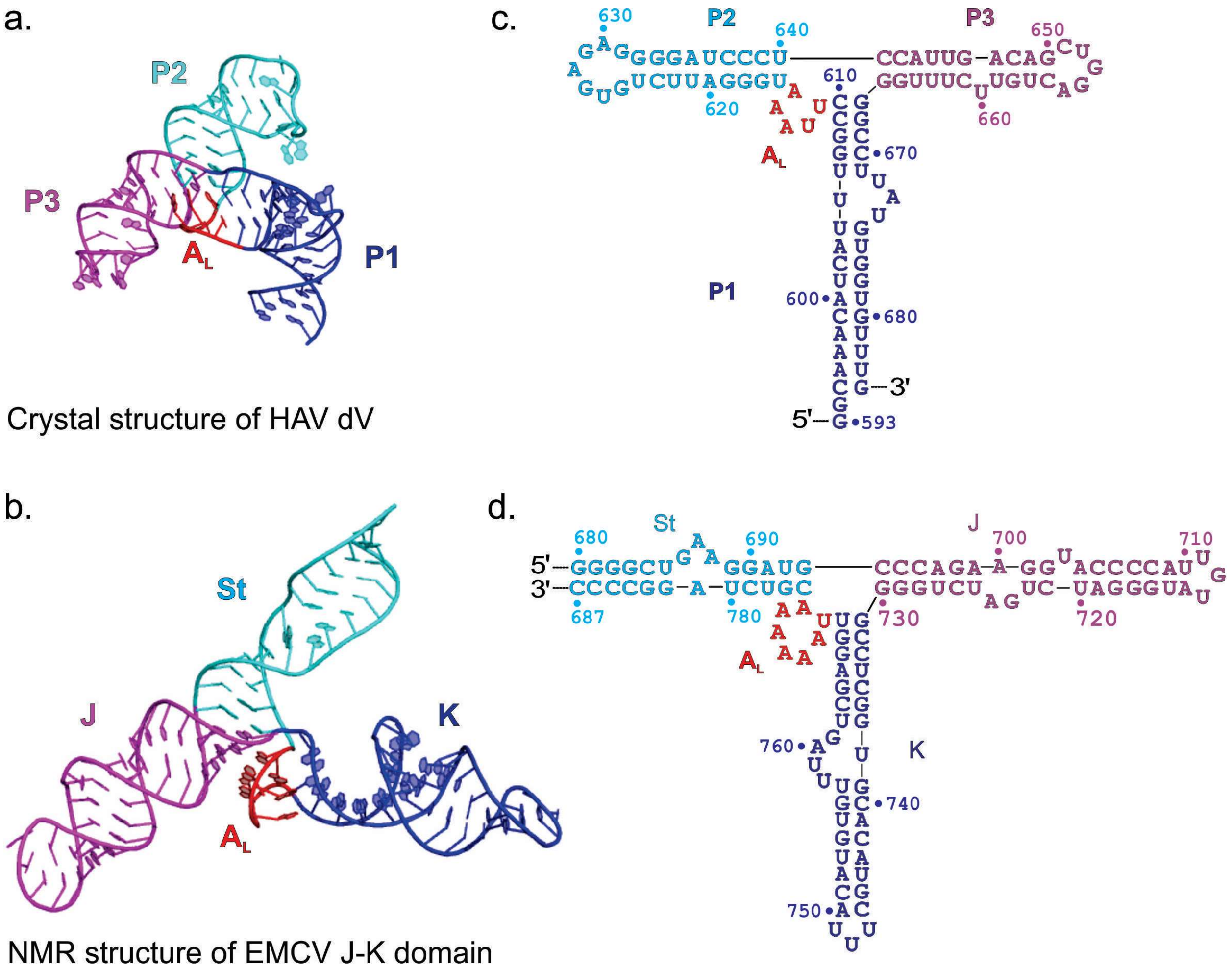

Supplementary Figure 13: Comparison between 3D structures of the HAV IRES dV and EMCV IRES J-K domain. (a) Crystal structure of HAV dV (this study). (b) Previously reported NMR structure of the EMCV J-K domain.[13] Secondary structures derived from respective 3D structures of HAV dV (c) and EMCV J-K domain (d). The EMCV J-K domain corresponds to a circularly permuted form of the HAV dV where the K, St, and J helices of EMCV J-K domain correspond to the P1, P2, and P3 helices of dV and 5' and 3'-ends reside at the base of St rather than P1. In both structures, an A_L motif organizes the topology of the corresponding three-way junction, and they contain an asymmetric bulge in analogous positions (helix P1 and K subdomain). The pre-organizing function of the A_L motif in the J-K domain facilitates binding of the heat domain of eIF4G between the St and K domains[13] and could function analogously in dV to facilitate interactions with eIF4G or other proteins.

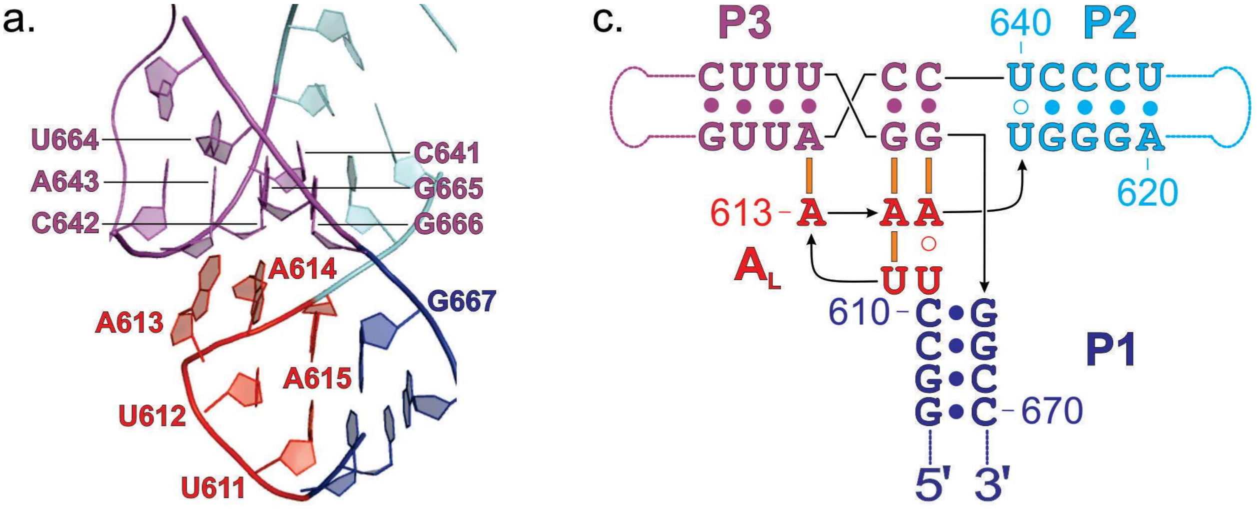

HAV dV three-way junction

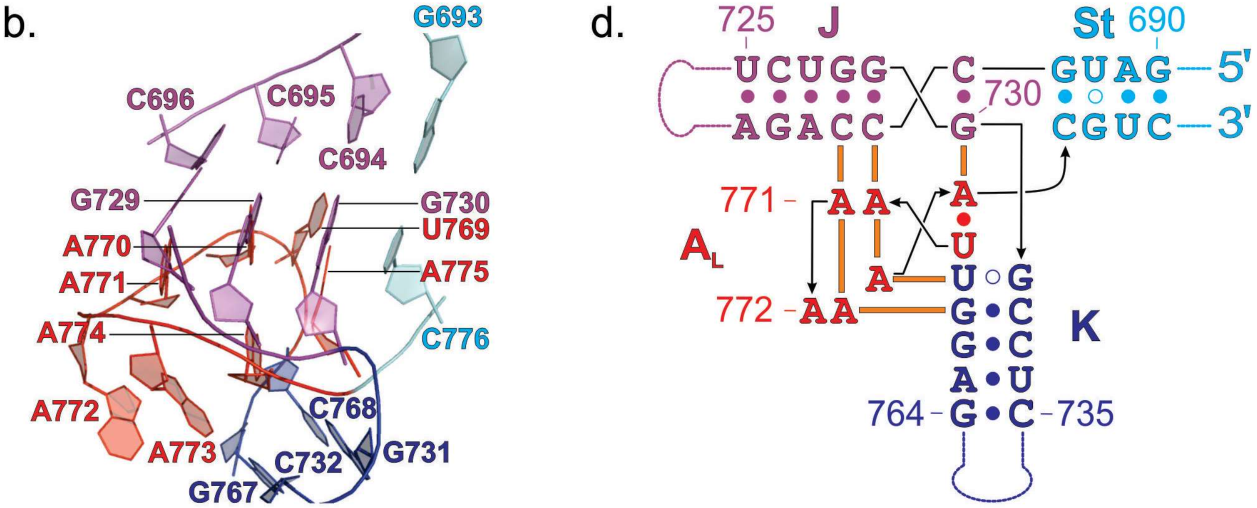

EMCV J-K domain three-way junction

Supplementary Figure 14: Comparison between the HAV dV and EMCV J-K domain three-way junctions. Overall structure of dV (a) and J-K domain (b) three-way junctions. Nucleotides engaged in secondary and tertiary interactions within the junctions are labelled. Secondary structures of (c) dV and (d) J-K domain showing tertiary interactions. Important regions and corresponding nucleotides are labelled and colored analogously in all panels. Solid and hollow circles in (c) and (d) indicate canonical and non-canonical interactions, respectively, and hollow bars represent the tertiary interactions. In these structures, although the exact identities of the interacting nucleotides are different, the global organization of the three-way junction and the A_L mediated interactions are strikingly similar.

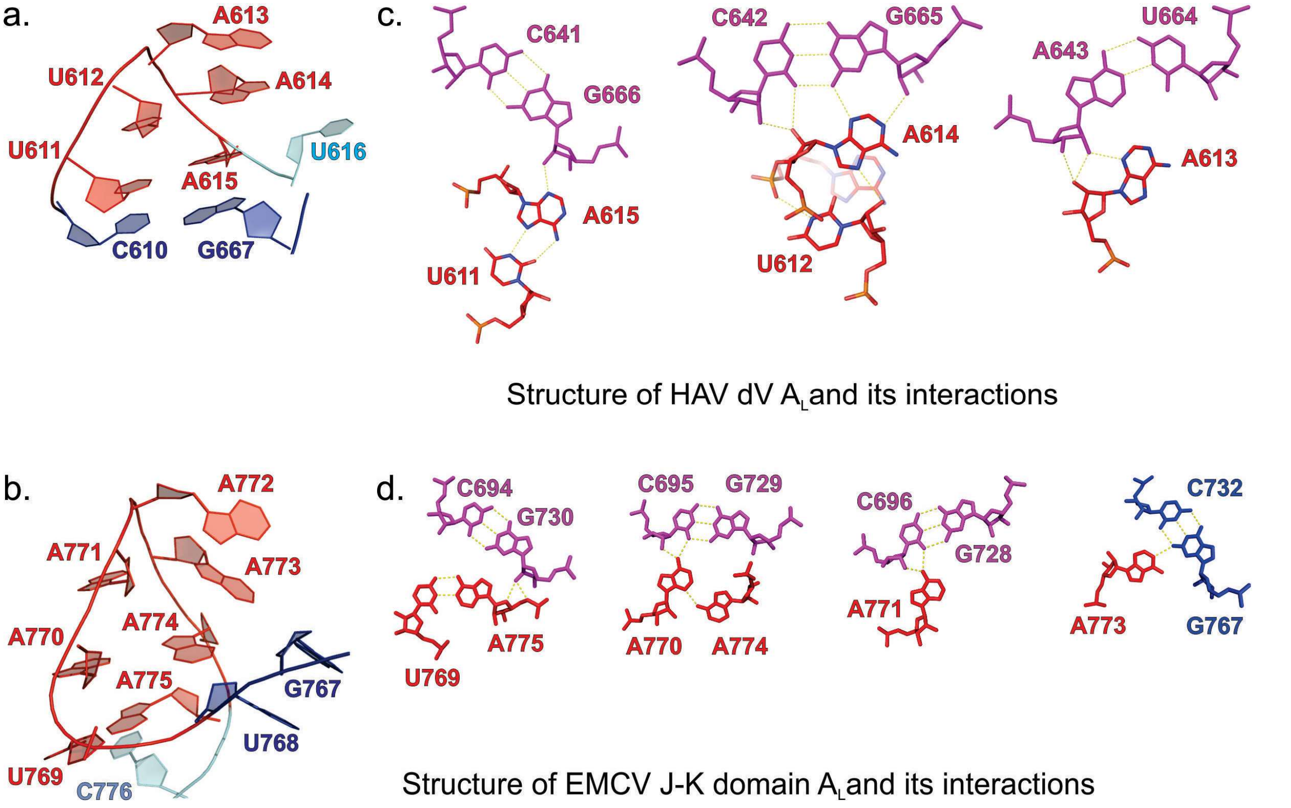

Supplementary Figure 15: Structure of A$_L$ motif and its interactions within the three-way junction. Global architecture of A$_L$ motif in HAV dV (a) and EMCV J-K domain (b). Nucleotides of A$_L$ motif are colored red. (c) and (d) Interactions of A$_L$ residues in the dV (c) and the J-K domain (d). In EMCV J-K domain, A$_L$ makes tertiary contacts with two, J and K helical stems. However, in HAV dV, it interacts only with the helical stem P3. Overall, the dV and the J-K domain three way-junctions are stabilized by forming three and four base triples, respectively. Yellow dashed lines indicate heteroatoms within hydrogen bonding distance (2.5 – 3.5 Å).

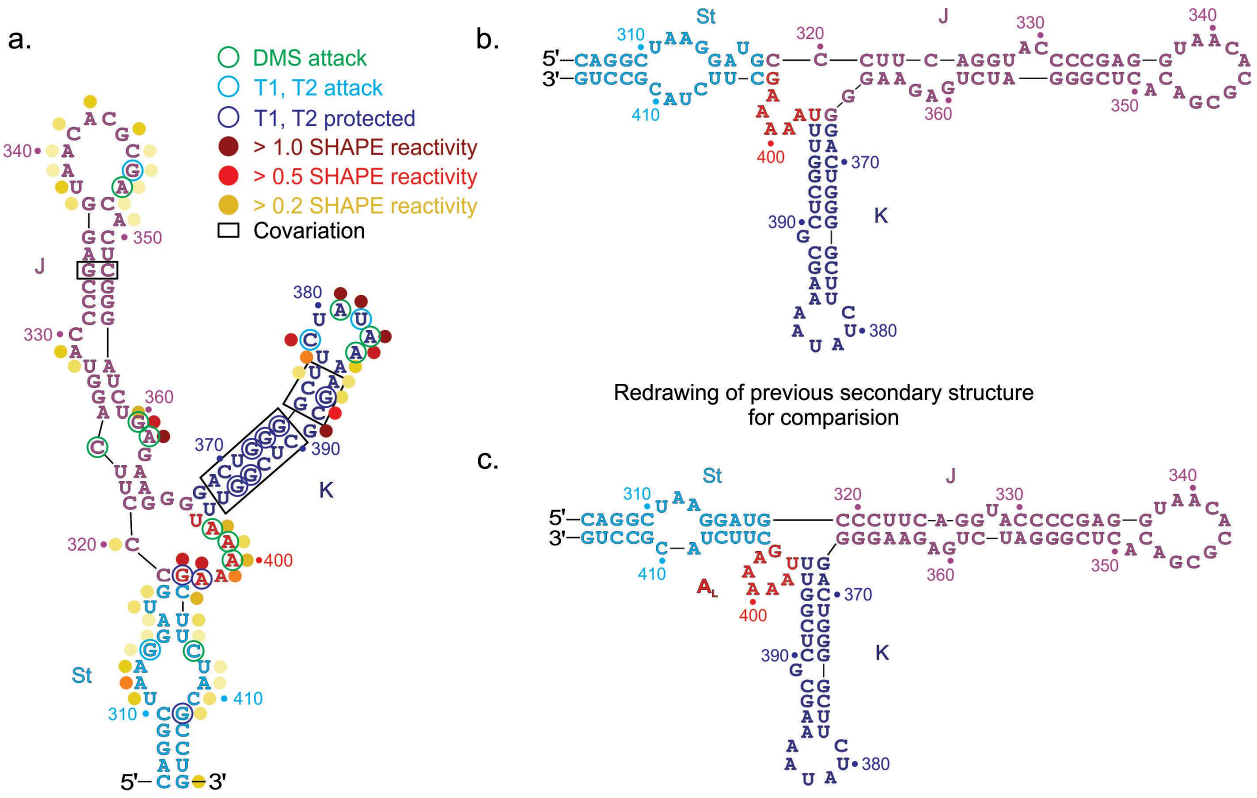

Previous RNA probing and co-variation analysis of FMDV IRES domain IV

Revised secondary structure model of FMDV dIV for *in silico* modeling based on its structural homology with EMCV J-K domain

Supplementary Figure 16: Proposed revision of FMDV IRES domain IV secondary structure based on analogous, high-resolution structures of HAV dV and EMCV J-K domain. (a) Secondary structure model of the FMDV domain IV based on previous biochemical analyses.[16] (b) Redrawing of the same model for convenient comparison. (c) Revised secondary structure of the FMDV domain IV based on its structural homology with EMCV J-K domain. The revised homology model, which predicts the formation of A_L motif within the three-way junction, was used for the computation of the FMDV dIV three-dimensional model. FMDV domain IV and EMCV J-K domain have ~50% sequence homology and biochemical analysis suggests that they adopt similar secondary structures that bind eIF4G in an analogous manner. The revised structure of domain IV improves its structural homology with the EMCV J-K domain (see Figure 5, Figure 6 and Supplementary Figure 11 for comparison) while satisfying the previous biochemical observations.[16] The A_L motif in the EMCV J-K domain and the FMDV domain IV both consist of five adenines within a pentaloop. However, a U○G wobble pair presumably closes the pentaloop in the domain IV rather than a canonical U•A pair in the J-K domain. By comparison, A_L of HAV dV consists of UAA trinucleotide loop closed by a Hoogsteen U•A pair.

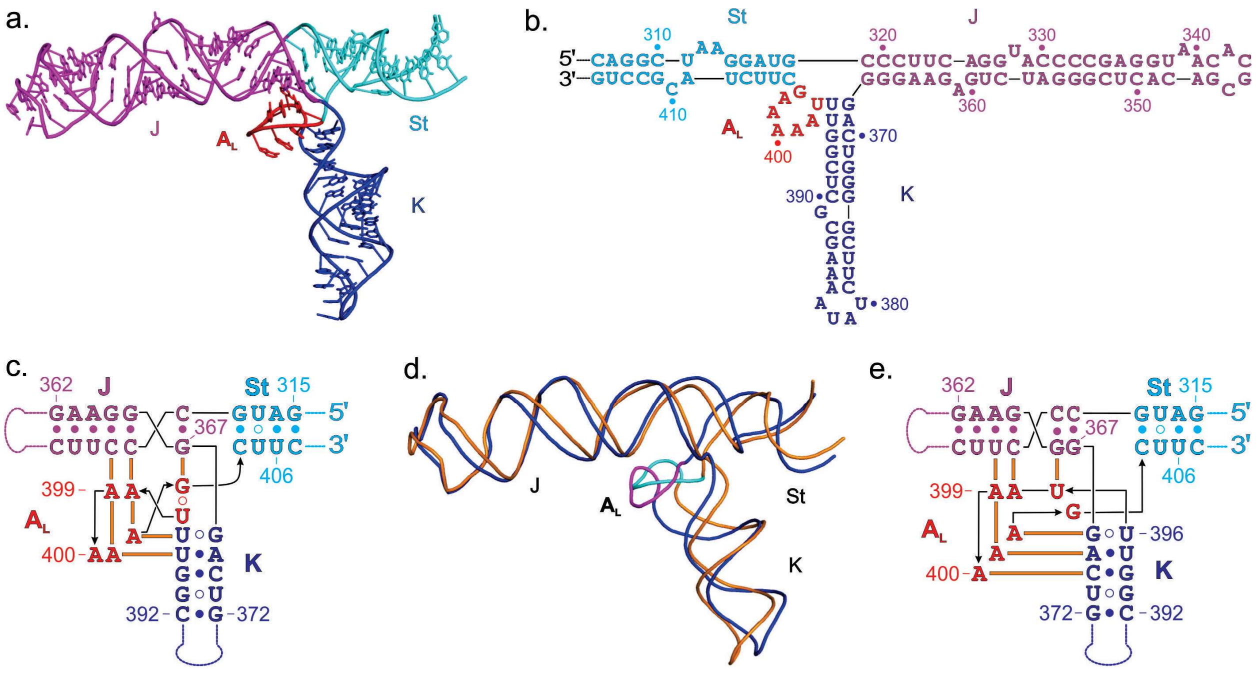

Supplementary Figure 17: Three-way junction structure of FMDV dIV. (a) *In silico* computed FMDV dIV model using the FARFAR and stepwise Monte Carlo Rosetta protocols[17,18] and (b), corresponding secondary structure. (c) Secondary and tertiary interactions within the dIV three-way junction. Solid and hollow circles depict canonical and non-canonical base-pairing interactions, respectively, and solid orange bars indicate the proposed tertiary interactions. As expected, the structure of FMDV domain IV highly resembles the EMCV J-K domain including the structure of A_L motif and overall three-way junction (see Figure 5, Figure 6 and Supplementary Figure 13-15 for comparison) and satisfies the previous biochemical observations.[16,19-21] In contrast to canonical U:A pair in EMCV J-K domain A_L, the FMDV dIV consist of a non-canonical U○G pair. One of the computed models without paring of U396 and G403 (d, blue) shows a slightly different configuration within the three-way junction (e) compared to that with U396○G403 pair (d, orange). For clarity, the A_L motif of the former is colored magenta and the later cyan. However, the overall secondary structure (shown in b) and the interactions of adenines, A398-A399 and A401-A402 with J and K helical stems, respectively, are almost identical in both cases. It is noteworthy that despite the prediction of a well-structured motif, the nucleotides predicted to form the A_L are not protected against the chemical modifications in biochemical probing assays (see Supplementary Figure 15). This is consistent with chemical probing for similar A-rich motifs in other RNA structures,[22,23] perhaps reflecting a dynamic character of this motif in solution.

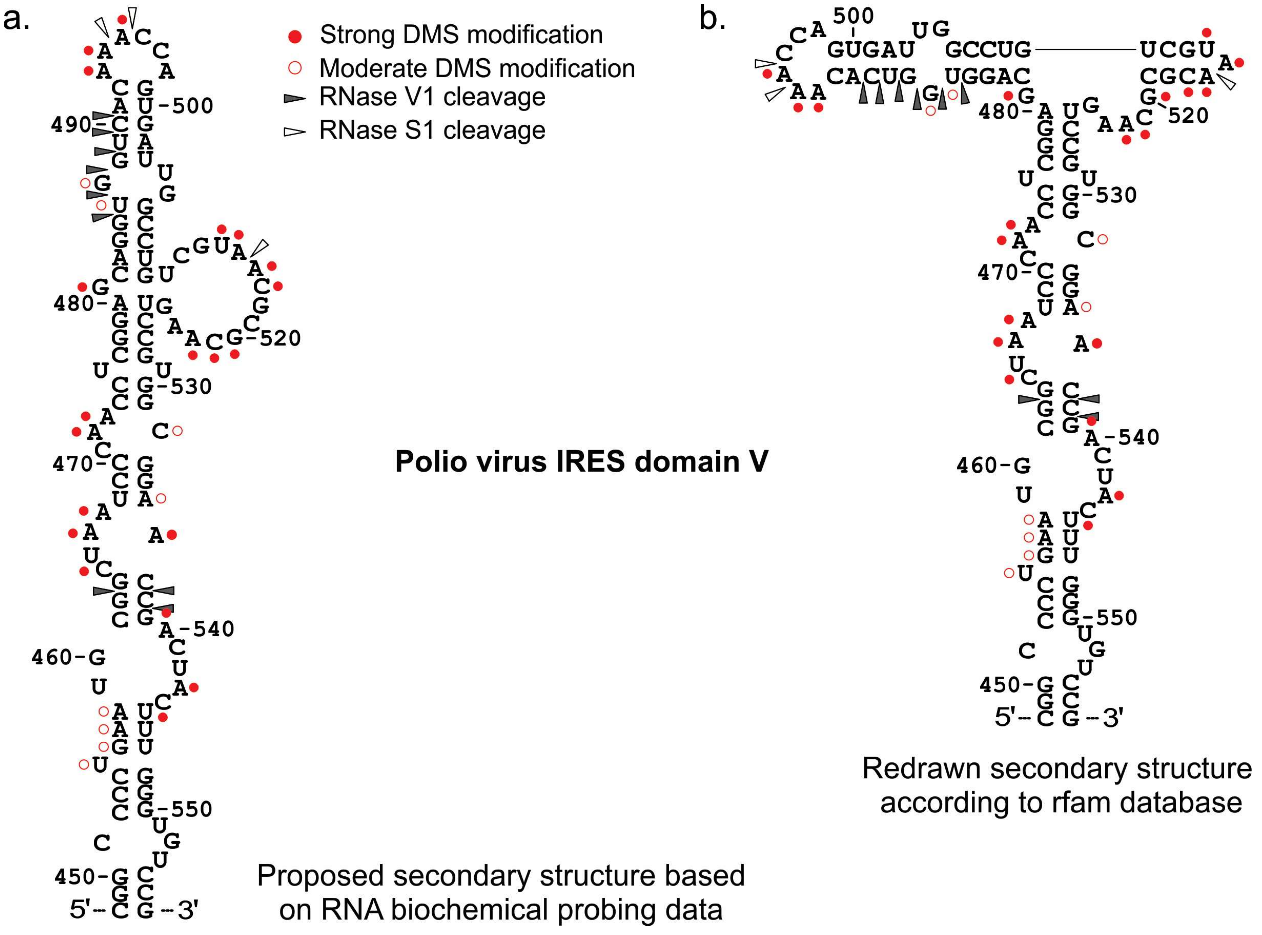

Supplementary Figure 18: Domain V of polio virus (PV) IRES (a type I picornaviral IRES) has a potential to form a three-way junction structure analogous to those observed for the HAV dV and the EMCV J-K domain. (a) Proposed secondary structure of PV domain V based on previous biochemical analysis.[19] (b) Secondary structure based on rfam bioinformatics database of picornaviral IRESs.[24] For comparison, probing data from (a) are also shown in (b). In the predicted structure, nucleotides 520-524 has the propensity to form an A_L type of motif to organize the corresponding three-way junction.

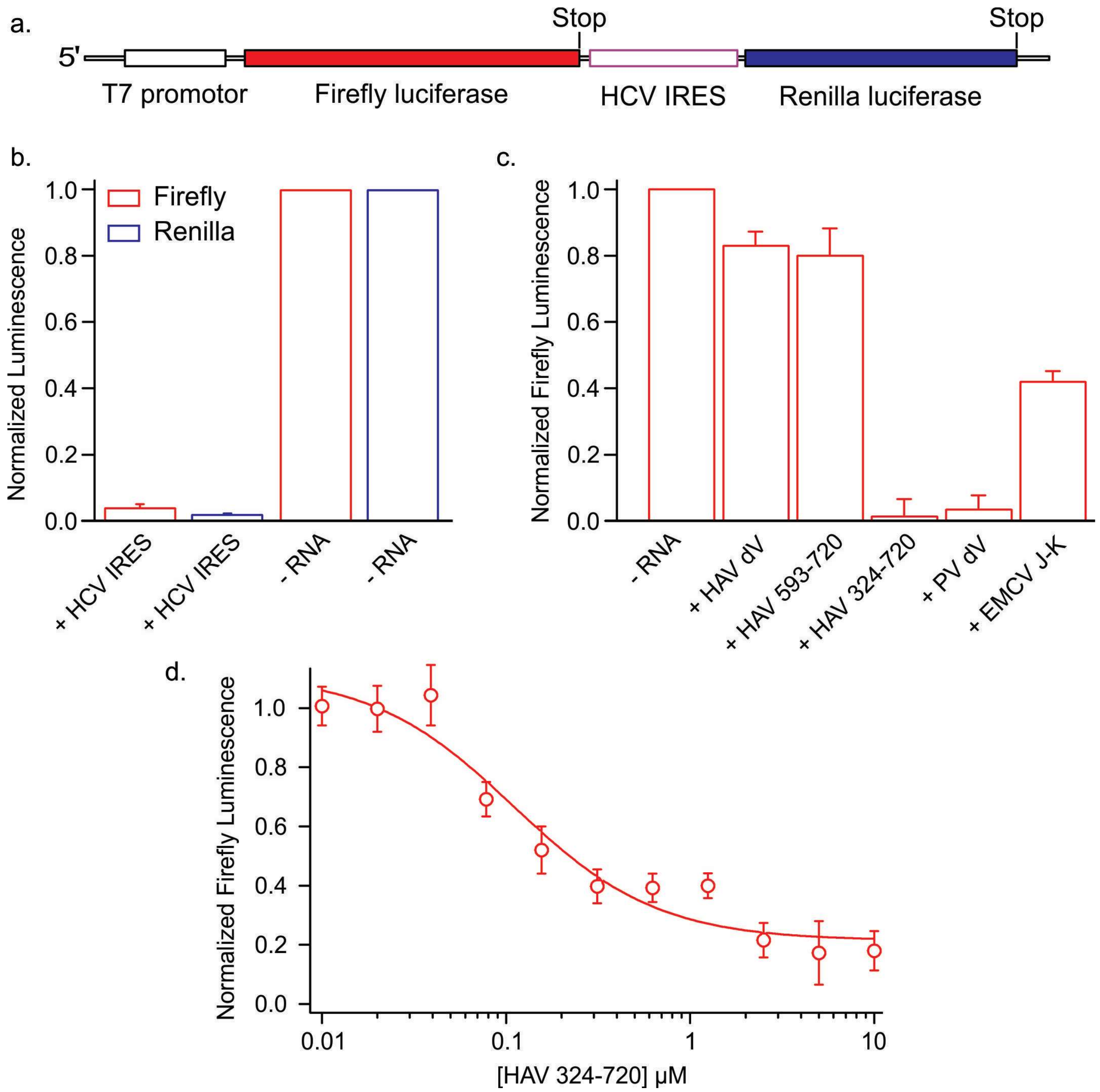

Supplementary Figure 19. *In vitro* translation of a bicistronic luciferase construct in rabbit reticulocyte lysate. (a) Design of the DNA template for the translation. The construct was generated via PCR of pFR_HCV_xb plasmid, which was a gift from Phillip A. Sharp (Addgene plasmid # 11510, http://www.addgene.org/11510/),[25] and the translation assay was performed by using a coupled transcription-translation kit (www.promega.com). In this assay, the firefly luciferase expression is controlled by canonical mechanism while renilla expression by hepatitis C virus (HCV) IRES. As HCV IRES binds 40S ribosome directly without requiring any translation initiation factors during translation initiation,[26-28] the added RNA constructs that can bind to the

40S ribosome would be expected to inhibit both firefly and renilla luciferase expression, whereas constructs that bind to eIFs (eIF4G or eIF4E or both) would be expected to inhibit the firefly luciferase expression, and possibly enhance firefly luciferase expression by eliminating competition with the upstream (firefly luciferase) open reading frame for ribosomes. The expression levels of the luciferases were detected by measuring the luminescence signals (Synergy Neo2 plate reader, www. biotek.com), which were obtained by using a dual-luciferase reporter assay (www.promega.com) (b) Normalized luminescence corresponding to firefly and renilla luciferase expression in the presence of HCV IRES RNA (2.5 μM, nts 40-372 of genotype 1b[27,29]) compared to that in the absence of any added RNA. (c) Luminescence corresponding to firefly luciferase expression normalized against the renilla luminescence in the presence of analogous RNA domains form HAV, PV and EMCV IRESs. Addition of IRES domains known to bind eIF4G (PV dV and EMCV J-K domain, 5 μM),[13,30] specifically inhibited firefly luciferase translation but not HCV IRES driven translation of renilla luciferase. In contrast, constructs corresponding to HAV dV alone (nts 593-684, 10 μM) or HAV dV plus dVI and a portion of polypyrimidine tract (HAV593-720, 10 μM) had little effect on translation of either luciferase. However, inclusion of domain IV yielded a construct (HAV324-720, 5 μM) that exhibited inhibition similar to the EMCV and PV constructs tested. (d) Suppression of firefly luminescence corresponding to the dose dependent inhibition of firefly luciferase expression by HAV 324 -720 construct with an IC50 of 110 ± 30 nM , consistent with the previous study that full length HAV IRES binds to eIF4G-4E complex with K_d of 94 ± 3 nM.[30] The error bars represent the standard deviations from three independent experiments.

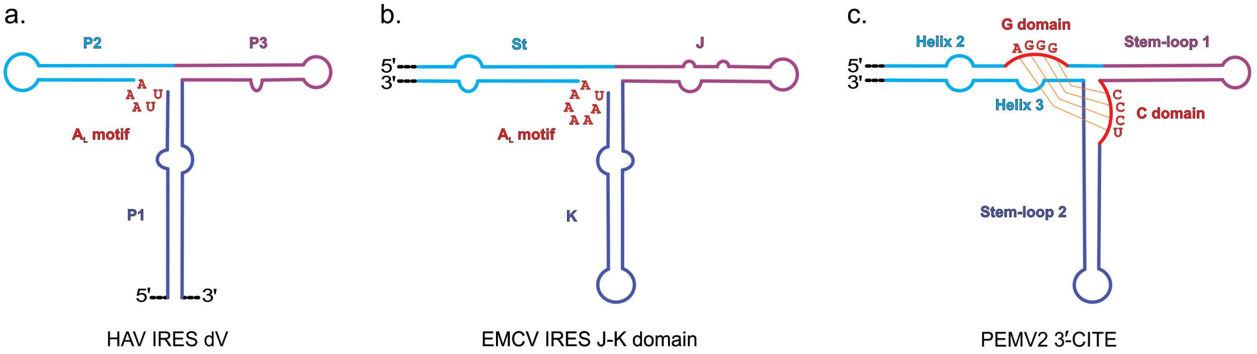

Supplementary Figure 20: Comparison of secondary structures of HAV IRES dV (a), EMCV IRES J-K domain (b) and PEMV2 3'-CITE (c). The secondary structures of the dV (crystal) and J-K domain (NMR)[13] were derived from the respective high-resolution structures, whereas PEMV2 3'-CITE represents a computationally calculated model.[31] Despite differences in sequence and strategy to organize the RNA structure, the overall 3-way junction topology of the EMCV J-K and PEMV2 3'-CITE is similar.

Supplementary References

1. Lu, D., Searles, M.A. & Klug, A. Crystal structure of a zinc-finger–RNA complex reveals two modes of molecular recognition. *Nature* **426**, 96 (2003).

2. Dominguez, D. et al. Sequence, Structure, and Context Preferences of Human RNA Binding Proteins. *Molecular cell* **70**, 854-867. e9 (2018).

3. Huang, L. & Lilley, D.M. The molecular recognition of kink-turn structure by the L7Ae class of proteins. *RNA* (2013).

4. Pham, V.V. et al. HIV-1 Tat interactions with cellular 7SK and viral TAR RNAs identifies dual structural mimicry. *Nature communications* **9**, 4266 (2018).

5. Cohen, J.I., Ticehurst, J.R., Purcell, R.H., Buckler-White, A. & Baroudy, B.M. Complete nucleotide sequence of wild-type hepatitis A virus: comparison with different strains of hepatitis A virus and other picornaviruses. *Journal of virology* **61**, 50-59 (1987).

6. Brown, E.A., Day, S.P., Jansen, R.W. & Lemon, S.M. The 5' nontranslated region of hepatitis A virus RNA: secondary structure and elements required for translation in vitro. *Journal of virology* **65**, 5828-5838 (1991).

7. Franke, D., Kikhney, A.G. & Svergun, D.I. Automated acquisition and analysis of small angle X-ray scattering data. *Nuc. Inst. Meth. A.* **689**, 52-59 (2012).

8. Schneidman-Duhovny, D., Hammel, M., Tainer, J.A. & Sali, A. Accurate SAXS profile computation and its assessment by contrast variation experiments. *Biophysical journal* **105**, 962-974 (2013).

9. Petoukhov, M.V., Konarev, P.V., Kikhney, A.G. & Svergun, D.I. ATSAS 2.1 – towards automated and web-supported small - angle scattering data analysis. *Journal of applied crystallography* **40**, s223-s228 (2007).

10. Svergun, D. Determination of the regularization parameter in indirect - transform methods using perceptual criteria. *Journal of applied crystallography* **25**, 495-503 (1992).

11. Franke, D. & Svergun, D.I. DAMMIF, a program for rapid ab-initio shape determination in small-angle scattering. *Journal of applied crystallography* **42**, 342-346 (2009).

12. Kozin, M.B. & Svergun, D.I. Automated matching of high-and low-resolution structural models. *Journal of applied crystallography* **34**, 33-41 (2001).

13. Imai, S., Kumar, P., Hellen, C.U., D'Souza, V.M. & Wagner, G. An accurately pre-organized IRES RNA structure enables eIF4G capture for initiating viral translation. *Nat. Struct. Mol. Biol.* **23**, 859 (2016).

14. Conn, G.L., Draper, D.E., Lattman, E.E. & Gittis, A.G. Crystal Structure of a Conserved Ribosomal Protein-RNA Complex. *Science* **284**, 1171-1174 (1999).

15. Ye, J.-D. et al. Synthetic antibodies for specific recognition and crystallization of structured RNA. *Proc. Natl. Acad. Sci. U.S.A.* **105**, 82-87 (2008).

16. Lozano, G. & Martínez-Salas, E. Structural insights into viral IRES-dependent translation mechanisms. *Current opinion in virology* **12**, 113-120 (2015).

17. Das, R., Karanicolas, J. & Baker, D. Atomic accuracy in predicting and designing noncanonical RNA structure. *Nature methods* **7**, 291 (2010).

18. Watkins, A.M. et al. Blind prediction of noncanonical RNA structure at atomic accuracy. *Science Advances* **4**, eaar5316 (2018).

19. Pilipenko, E.V. et al. Conserved structural domains in the 5′ -untranslated region of picornaviral genomes: an analysis of the segment controlling translation and neurovirulence. *Virology* **168**, 201-209 (1989).

20. Pilipenko, E., Blinov, V., Chernov, B., Dmitrieva, T. & Agol, V. Conservation of the secondary structure elements of the 5'untranslated region of cardio-and aphthovirus RNAs. *Nucleic acids research* **17**, 5701-5711 (1989).

21. Pilipenko, E.V. et al. A cell cycle-dependent protein serves as a template-specific translation initiation factor. *Genes & Development* **14**, 2028-2045 (2000).

22. Uchiumi, T., Honma, S., Endo, Y. & Hachimori, A. Ribosomal proteins at the stalk region modulate functional rRNA structures in the GTPase center. *Journal of Biological Chemistry* **277**, 41401-41409 (2002).

23. Rosendahl, G. & Douthwaite, S. Ribosomal proteins L11 and L10.(L12) 4 and the antibiotic thiostrepton interact with overlapping regions of the 23 S rRNA backbone in the ribosomal GTPase centre. *Journal of molecular biology* **234**, 1013-1020 (1993).

24. Kalvari, I. et al. Rfam 13.0: shifting to a genome-centric resource for non-coding RNA families. *Nucleic acids research* **46**, D335-D342 (2018).

25. Petersen, C.P., Bordeleau, M.-E., Pelletier, J. & Sharp, P.A. Short RNAs repress translation after initiation in mammalian cells. *Molecular cell* **21**, 533-542 (2006).

26. Fraser, C.S. & Doudna, J.A. Structural and mechanistic insights into hepatitis C viral translation initiation. *Nature Reviews Microbiology* **5**, 29-38 (2007).

27. KIEFT, J.S., ZHOU, K., JUBIN, R. & DOUDNA, J.A. Mechanism of ribosome recruitment by hepatitis C IRES RNA. *Rna* **7**, 194-206 (2001).

28. Otto, G.A. & Puglisi, J.D. The pathway of HCV IRES-mediated translation initiation. *Cell* **119**, 369-380 (2004).

29. Brown, E.A., Zhang, H., Ping, L.-H. & Lemon, S.M. Secondary structure of the 5′ nontranslated regions of hepatitis C virus and pestivirus genomic RNAs. *Nucleic acids research* **20**, 5041-5045 (1992).

30. Avanzino, B.C., Fuchs, G. & Fraser, C.S. Cellular cap-binding protein, eIF4E, promotes picornavirus genome restructuring and translation. *Proceedings of the National Academy of Sciences* **114**, 9611-9616 (2017).

31. Wang, Z., Parisien, M., Scheets, K. & Miller, W.A. The cap-binding translation initiation factor, eIF4E, binds a pseudoknot in a viral cap-independent translation element. *Structure* **19**, 868-880 (2011).
