# Supplementary material for: A conserved RNA structural motif for organizing topology within picornaviral internal ribosome entry sites
Source: Nat Commun. 2019 Aug 9;10:3629. doi: 10.1038/s41467-019-11585-z (PMC6689051; doi:10.1038/s41467-019-11585-z)
Supplement: Supplementary file 3 — Reporting Summary [file 41467_2019_11585_MOESM3_ESM.pdf]

nature research

Corresponding author(s): Joseph A. Piccirilli
NCOMMS-18-37955A

Last updated by author(s): Jun 19, 2019

Reporting Summary

Nature Research wishes to improve the reproducibility of the work that we publish. This form provides structure for consistency and transparency in reporting. For further information on Nature Research policies, see Authors & Referees and the Editorial Policy Checklist.

Statistics

For all statistical analyses, confirm that the following items are present in the figure legend, table legend, main text, or Methods section.

n/a	Confirmed	
☐	☒	The exact sample size (n) for each experimental group/condition, given as a discrete number and unit of measurement
☐	☒	A statement on whether measurements were taken from distinct samples or whether the same sample was measured repeatedly
☒	☐	The statistical test(s) used AND whether they are one- or two-sided<br>*Only common tests should be described solely by name; describe more complex techniques in the Methods section.*
☒	☐	A description of all covariates tested
☐	☒	A description of any assumptions or corrections, such as tests of normality and adjustment for multiple comparisons
☐	☒	A full description of the statistical parameters including central tendency (e.g. means) or other basic estimates (e.g. regression coefficient) AND variation (e.g. standard deviation) or associated estimates of uncertainty (e.g. confidence intervals)
☒	☐	For null hypothesis testing, the test statistic (e.g. F, t, r) with confidence intervals, effect sizes, degrees of freedom and P value noted<br>*Give P values as exact values whenever suitable.*
☒	☐	For Bayesian analysis, information on the choice of priors and Markov chain Monte Carlo settings
☒	☐	For hierarchical and complex designs, identification of the appropriate level for tests and full reporting of outcomes
☒	☐	Estimates of effect sizes (e.g. Cohen's d, Pearson's r), indicating how they were calculated

Our web collection on statistics for biologists contains articles on many of the points above.

Software and code

Policy information about availability of computer code

Data collection	Details of data collection strategy are provided in the methods section. X-ray crystallographic data were collected at Argonne National Laboratory beam-line 24-ID-B and 24-ID-C. SAXS data were collected at Advanced Light Source beam-line SIBYLS, a US DOE Office of Science User Facility operated for the DOE Office of Science by Lawrence Berkeley National Laboratory.
Data analysis	Details of data analysis software are provided in the methods section.

For manuscripts utilizing custom algorithms or software that are central to the research but not yet described in published literature, software must be made available to editors/reviewers. We strongly encourage code deposition in a community repository (e.g. GitHub). See the Nature Research guidelines for submitting code & software for further information.

Data

Policy information about availability of data

All manuscripts must include a data availability statement. This statement should provide the following information, where applicable:

- Accession codes, unique identifiers, or web links for publicly available datasets
- A list of figures that have associated raw data
- A description of any restrictions on data availability

Purified Fab HAVx or the plasmid for Fab expression will be provided upon request. Request should be addressed to Joseph A. Piccirilli. Details of the Fab expression and purification protocols have been provided in the methods section. Atomic coordinates and structure factors for the reported crystal structure have been deposited with the Protein Data Bank under accession number 6MWN. All input files and scripts necessary to conduct the modeling are provided as GitHub repository as described in methods section.

Field-specific reporting

Please select the one below that is the best fit for your research. If you are not sure, read the appropriate sections before making your selection.

☒ Life sciences ☐ Behavioural & social sciences ☐ Ecological, evolutionary & environmental sciences

For a reference copy of the document with all sections, see nature.com/documents/nr-reporting-summary-flat.pdf

Life sciences study design

All studies must disclose on these points even when the disclosure is negative.

Sample size	No statistical methods were used to determine the sample size. The sample sizes chosen for each experiment that were sufficient to support the conclusions. Sample sizes are indicated in the figure legends wherever applicable.
Data exclusions	No data was excluded from the analysis except from the failed experiments. Reflection outliers of crystallographic dataset were automatically excluded using established programs and criteria.
Replication	All attempts to reproduce the datasets were successful.
Randomization	Not relevant to the study.
Blinding	Authors were not aware of the outcomes of the experiments.

Reporting for specific materials, systems and methods

We require information from authors about some types of materials, experimental systems and methods used in many studies. Here, indicate whether each material, system or method listed is relevant to your study. If you are not sure if a list item applies to your research, read the appropriate section before selecting a response.

Materials & experimental systems

n/a	Involved in the study	
☐	☒	Antibodies
☒	☐	Eukaryotic cell lines
☒	☐	Palaeontology
☒	☐	Animals and other organisms
☒	☐	Human research participants
☒	☐	Clinical data

Methods

n/a	Involved in the study	
☒	☐	ChIP-seq
☒	☐	Flow cytometry
☒	☐	MRI-based neuroimaging

Antibodies

Antibodies used	Synthetic anti-RNA antibodies.
Validation	Details about the antibodies used in this study can be found in the methods section.
